# Stimulus Incongruency Effects on Visual Short-Term Memory Binding in Young and Older Adults with and Without Cognitive Impairment

**DOI:** 10.3390/brainsci16080898

**Published:** 2026-08-21

**Authors:** Monika McAtarsney-Kovacs, Raju Sapkota, Ian van der Linde, Shahina Pardhan

**Affiliations:** 1 Vision & Eye Research Institute, School of Medicine, Anglia Ruskin University, Cambridge CB1 1PT, UK; monika.mcatarsney-kovacs@aru.ac.uk (M.M.-K.); raju.sapkota@aru.ac.uk (R.S.); ian.vanderlinde@aru.ac.uk (I.v.d.L.); 2 School of Computing and Information Science, Anglia Ruskin University, Cambridge CB1 1PT, UK; 3 Cognition and Neuroscience Group, ARU Centre for Mind and Behaviour, Anglia Ruskin University, Cambridge CB1 1PT, UK

**Keywords:** visual short-term memory, Stroop effect, mild cognitive impairment, congruency, directional binding, memory accuracy, reaction time

## Abstract

**Highlights:**

**What are the main findings?**

- Word-location binding for Stroop-like stimuli during a visual short-term memory (VSTM) task revealed that incongruent color-word stimuli (e.g., the word “RED” printed in a non-red ink) produced significantly longer reaction times compared to congruent stimuli (e.g., the word “RED” printed in red ink) in cognitively normal older adults and older adults screening positive for mild cognitive impairment (MCI), but not in young adults. When averaged across congruent and incongruent trials reaction time increased and memory accuracy decreased significantly for adults screening positive for MCI compared to the cognitively normal older adults and young healthy adults.
- Incongruent trials in the directional binding task (i.e., between the direction of pointing and the movement of an arrow) resulted in longer reaction times than incongruent trials for both young and cognitively healthy older adults, not for adults screening positive for MCI. Individuals screening positive for MCI performed worst overall regardless of the congruency condition.

**What are the implications of the main findings?**

- Reaction time measures during VSTM binding tasks involving incongruent stimuli appear to capture variance not reflected in accuracy measures.
- Incorporating Stroop-like interference into VSTM binding tasks has the potential to enhance early detection of MCI and should be evaluated further in clinically diagnosed MCI cohorts.

**Abstract:**

Background: Literature suggests that both the Stroop task and visual short-term memory (VSTM) binding tasks can differentiate individuals with Mild Cognitive Impairment (MCI) from cognitively normal older adults. These cognitive processes have rarely been examined together. This study investigated how Stroop-like color-word incongruency and directional incongruency between arrow orientation and motion affect memory accuracy and reaction time (RT) measures during a VSTM binding task in cognitively normal adults and individuals screening positive for MCI. Methods: A total of 115 adults participated: 34 healthy young adults (mean age = 24.7 ± 3.0 years), 61 cognitively normal older adults (mean age = 70.5 ± 6.3 years), and 20 older adults screening positive for MCI (mean age = 73.9 ± 5.9 years), i.e., MCI-indicatives. Participants completed two non-speeded VSTM binding tasks under low (two items) and high (four items) memory load conditions. Experiment 1 used color words displayed in congruent or incongruent ink colors, while Experiment 2 used arrows whose orientation and motion directions were congruent or incongruent. Results: In Experiment 1, overall % memory accuracy was significantly lower in the MCI-indicative group than in the cognitively normal older group and the young healthy group (*p* < 0.001); the difference between the young and cognitively normal older group was, however, not significant. In Experiment 2, % memory accuracy in the cognitively normal older group was significantly lower than in the young group but better than in the MCI-indicative group (*p* < 0.01). Across both experiments, the MCI-indicative group showed slower RTs overall. Incongruency did not affect accuracy but significantly increased RTs in older adults and the MCI group in Experiment 1 (*p* < 0.001), with no significant effect in young adults. Conclusions: The MCI-indicative group showed significantly decreased overall accuracy and longer RTs during VSTM tasks involving Stroop-like colored word-location and directional binding. While Stroop-like incongruency and directional incongruency did not affect memory accuracy, they selectively increased RT in older and MCI-indicative groups, suggesting that executive processing demands may uncover subtle cognitive changes associated with normal aging and early MCI.

## 1. Introduction

Visual short-term memory (VSTM) stores visual information, such as color, shape, and spatial location or a combination of these features, for a few seconds before the information is forgotten or consolidated into long-term memory [1,2,3,4]. VSTM supports a wide range of everyday activities, including scene perception, spatial navigation, visual search, and change detection [1,5,6].

Opinions differ regarding the mechanisms by which information is stored in VSTM. According to the Slot Model, objects are stored in VSTM within a fixed number of discrete slots [7], typically around four, depending on stimulus familiarity, similarity, complexity, and spatial layout [2,8]. The Resource Model proposes that VSTM is a shared resource with no upper limit in the number of objects that can be stored, and as the number of objects or their complexity increases, the precision with which they are stored in VSTM decreases [9,10].

The precision of VSTM representations may also depend upon how the stimulus features are related to each other: specifically, whether those features share perceptual or semantic characteristics (congruent) or differ across those dimensions (incongruent). In an object-only memory recognition task (where location was irrelevant), performance was shown to be significantly impaired when the target object appeared at a different (incongruent) location than where it was originally seen [11]. Similarly, as seen in the classic Stroop task, when there is incongruency between the color name and the ink color in which the name is printed (e.g., the word “RED” in blue ink), identifying the ink color becomes more difficult compared to when the color name is printed in the same ink (e.g., “RED” in red ink) resulting in slower response times and increased error rates [12,13].

Schema-based theory, a framework for explaining how the human brain structures and processes information, proposes that schema-consistent information arising from stimulus congruency is encoded more efficiently in the brain [14,15,16,17,18,19,20,21,22]. According to the theory of inhibition control, older adults are less able to inhibit task-irrelevant information [23,24,25,26,27,28], which has been associated with structural and functional changes in the anterior cingulate and prefrontal cortices, the brain areas responsible for conflict monitoring and attentional control [23,24,29]. Notably, these brain areas are also affected in mild cognitive impairment (MCI) and prodromal Alzheimer’s disease [25,26,27,28].

While individuals with MCI may be expected to experience greater difficulty in resolving conflicting information arising from the stimulus incongruency [30,31,32,33,34,35], the magnitude and nature of such effects may also depend upon whether during the memory processing stage participants would be aware of incongruent (task-irrelevant) information. Specifically, the impact of stimulus incongruency may differ depending upon whether incongruent features are known from the outset during encoding or become apparent only at retrieval. The present study was therefore designed to examine these alternative possibilities. It is also noteworthy that, although both the Stroop effect and age-related VSTM decline involve prefrontal cortex dysfunction, these have largely been studied independently in individuals with MCI, despite the prefrontal cortex also being one of the brain regions affected in MCI [36,37].

The above-mentioned knowledge gaps were addressed in the present study, in which healthy young adults, cognitively normal older adults and older adults screening positive for MCI completed two VSTM experiments (non-speeded). In Experiment 1 (color word-location binding), Stroop-like stimuli were used, and participants were explicitly instructed from the outset that color information was task-irrelevant and may be ignored. Experiment 2 (directional binding) examined how conflict between the orientation and the direction of motion of drifting arrows influences VSTM performance. Participants were not informed about the task-irrelevant stimulus feature prior to test display. Results indicate that although the tasks were non-speeded, stimulus incongruency significantly affected reaction times (RTs), with this effect being more significant in adults screening positive for MCI than in cognitively normal older adults and healthy young adults. Our findings suggest that RT measures may capture variance not reflected in accuracy measures and warrant further evaluation.

## 2. Materials and Methods

### 2.1. Participants

A total of 115 participants were recruited. Thirty-four healthy young adults (aged 18–30 years, 56% female, mean years of education 18), 61 normally aging older adults (aged ≥ 55 years, 54% female, mean years of education 16.9), and 20 older adults screening positive for MCI (aged ≥ 55 years, 35% female, mean years of education 16.5) were recruited through the Joint Dementia Research platform, the University of the Third Age (U3A), and by poster and flyer advertisements at Anglia Ruskin University, and a local retirement home. The group screened positive for MCI included older adults who met the subthreshold criteria of either the MoCA (scored < 26 after applying the standard +1 education adjustment for individuals with ≤12 years of education) or/and of the ACE-III (scored < 88). Our sample size of the MCI-indicative group is similar to or greater than some of the previous studies [38,39,40]. Furthermore, recruitment of MCI and dementia individuals is a well-recognized challenge in cognitive research [41]. Healthy older adults had higher MoCA (M = 28, SD = 1.28) and ACE-III scores (M = 96, SD = 2.95) compared to older adults screening positive for MCI (MoCA: M = 24, SD = 3.12; ACE-III: M = 88, SD = 10.31), hereafter referred to as MCI-indicatives. All participants also completed Experiment 2; however, one participant in the healthy young group did not complete Experiment 1. Consequently, analyses for Experiment 1 were conducted with 33 young adults.

To ensure that participants had normal or corrected-to-normal visual acuity and color vision, all participants went through visual acuity (MNREAD Acuity Chart 2) [42] and color vision (City University Color Vision Test, 3rd Edition 1998) [43] tests at the beginning of the assessment. All participants demonstrated normal or corrected-to-normal visual acuity (≥6/6, equivalent to normal vision) and color vision. Exclusion criteria comprised the following self-reported conditions: uncorrected visual and hearing impairment, known history of neurological or psychological disorders (excluding MCI), brain injury, epilepsy, dyslexia, stroke, degenerative diseases such as Parkinson’s, depression, anxiety, and chronic alcohol and drug use.

### 2.2. Stimuli and Apparatus

**Figure 1:**
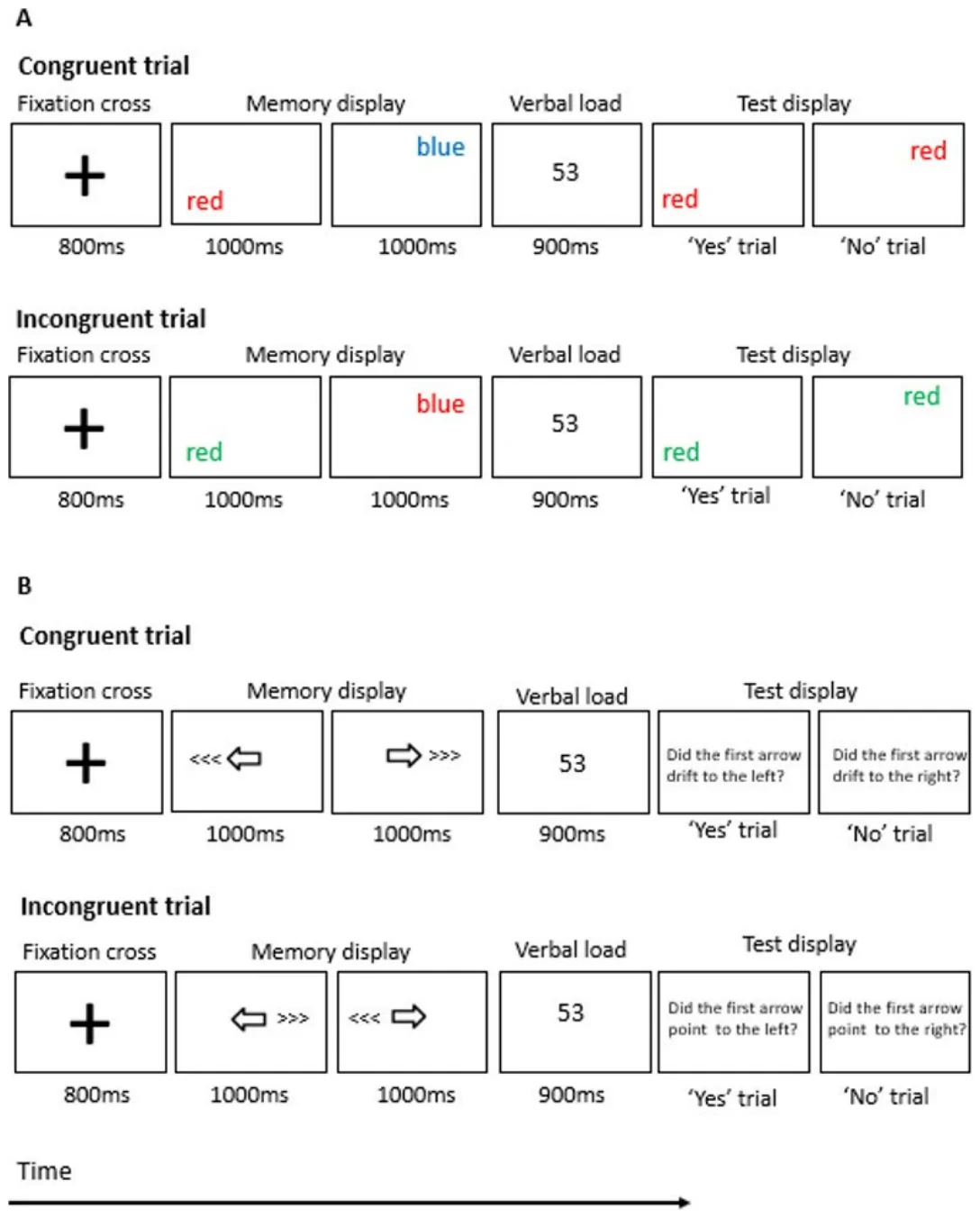
Schematic diagram of the experimental procedure (with ML2), and the type of stimuli used in Experiment 1 (**A**) and Experiment 2 (**B**).

Stimuli comprised Stroop-like colored words (Experiment 1) and drifting arrows (Experiment 2) as shown in Figure 1. Stimuli were displayed using a laptop computer (Dell Precision, M4500, TX, USA) connected to an external display (Sony, GDM-F520, Tokyo, Japan). The screen resolution was set at 1440 × 900 pixels with a refresh rate of 60 Hz.

A chin and forehead rest was used to maintain participants’ viewing position and distance, which was approximately 60 cm from the display screen. Participants responded yes or no to the task using the left and right buttons of a mouse (Logitech M185, Logitech, Suzhou, China) placed in their dominant hand. Visual cues (yes and no stickers on the right and left buttons of the mouse, respectively) reminded participants of which button to press. Participant responses were automatically stored by the computer and categorized according to the signal detection theory [44]. The experiments were implemented in SR Research Experiment Builder software version 2.3.38. (SR Research Ltd., Ottawa, ON, Canada).

### 2.3. Experimental Procedure

#### 2.3.1. Experiment 1

Figure 1A provides the schematic diagram of the experimental procedure used in Experiment 1. Each trial began with a fixation cross presented at the display screen center for 800 ms. Next, either two (memory load 2; ML2) or four (memory load 4; ML4) stimuli were presented sequentially (each for 1000 ms) at any of the 64 computer-generated random locations within a 700 × 700-pixel grid centered on the memory display screen. Stimuli consisted of color words with either complementary (congruent) or contradictory (incongruent) ink colors. This was followed by the presentation of a random two-digit number for 900 ms that participants had to read out loud to discourage verbal retention of the stimuli and recruitment of long-term memory support (articulatory suppression) [45,46]. Before and after the presentation of the two-digit number, a blank screen was presented for 550 ms.

Participants were told that they could ignore the ink color of the words (hence a Stroop-like task) but were required to remember the word and its location, and to respond to the question ‘Was this color word displayed here?’. During the test display, a previously presented color word from the memory display appeared in the same location (‘yes’ trial) or in the location of another color word (‘no’ trial).

The experiment consisted of 16 trials, divided equally between memory loads (ML2 and ML4) and trial type (‘yes’ and ‘no’ trials). There were four practice trials at the beginning of the test that were discounted.

#### 2.3.2. Experiment 2

Figure 1B provides the schematic diagram of the procedure used in Experiment 2. Each trial began with a fixation cross displayed at the center of the screen for 800 ms. Next, either two (ML2) or four arrows (ML4) were presented sequentially, each drifting and pointing either to the right or to the left. The starting point of the drifting arrows was at the center of the screen. In congruent trials, arrows were drifting and pointing in the same direction, while in incongruent trials, arrows were drifting in the opposite direction of their pointing direction. This was followed by the presentation of a random two-digit number for 900 ms that participants had to read out loud to discourage verbal labelling of the stimuli. Before and after the presentation of the two-digit number, a blank screen was presented for 550 ms. Next, a test display was presented, and participants were requested to answer the question pertaining to the pointing or drifting direction of one of the arrows in the memory display. Participants were required to remember the direction of both the drifting and pointing of the arrow and respond to the questions, ‘Did the second arrow point to the left?’ or ‘Did the first arrow drift to the right?’ Across trials, questions referred equally often to the first and second arrows (in ML4 to the first, second, third, and fourth), and equally often to pointing and drift directions. Participants responded either yes/no using the left and right buttons of a mouse. The next trial commenced immediately after a response was submitted. The test consisted of eight trials with ML2 and 32 trials with ML4, divided equally between congruent and incongruent and yes and no trials. There were four practice trials at the beginning of the test.

While both experiments were self-paced and participants were explicitly instructed to focus on accuracy rather than speed, RT data were also collected and are reported alongside accuracy data. To alleviate symptoms of fatigue, participants could take frequent rest breaks by informing the experimenter. The task was non-speeded in that participants were not subject to time pressure to respond. Nonetheless, RT measures remain an informative behavioral measure, reflecting the duration of the cognitive processes required to perform the task. RT data are often reported with percentage accuracy data for non-speeded cognitive tasks precisely for this reason [47]. In such contexts, RT is not interpreted as a measure of speeded performance per se, but rather as an index of the cognitive processing demands associated with task completion.

### 2.4. Statistical Methods

Both accuracy (% correct responses) and the RT data were analyzed. All statistical analyses were performed in IBM SPSS Statistics version 31.0.0.0, using a 3 (groups) × 2 (memory load) × 2 (congruency type) mixed ANOVA. Post hoc pairwise comparisons were conducted using Bonferroni-corrected tests to control the family-wise error rate. Where Levene’s test indicated that the assumption of homogeneity of variance was violated (*p* < 0.05), Welch’s ANOVA was used with the Games–Howell post hoc test as a follow-up analysis for group effects.

A sensitivity analysis was conducted using G*Power 3.1 [48] for a repeated-measures within–between interaction. Assuming α = 0.05 and power = 0.80, the available sample size (*N* = 115) was sufficient to detect effects of Cohen’s f = 0.12 or larger.

## 3. Results

### 3.1. Experiment 1

#### 3.1.1. Memory Accuracy (% Correct Responses)

**Figure 2:**
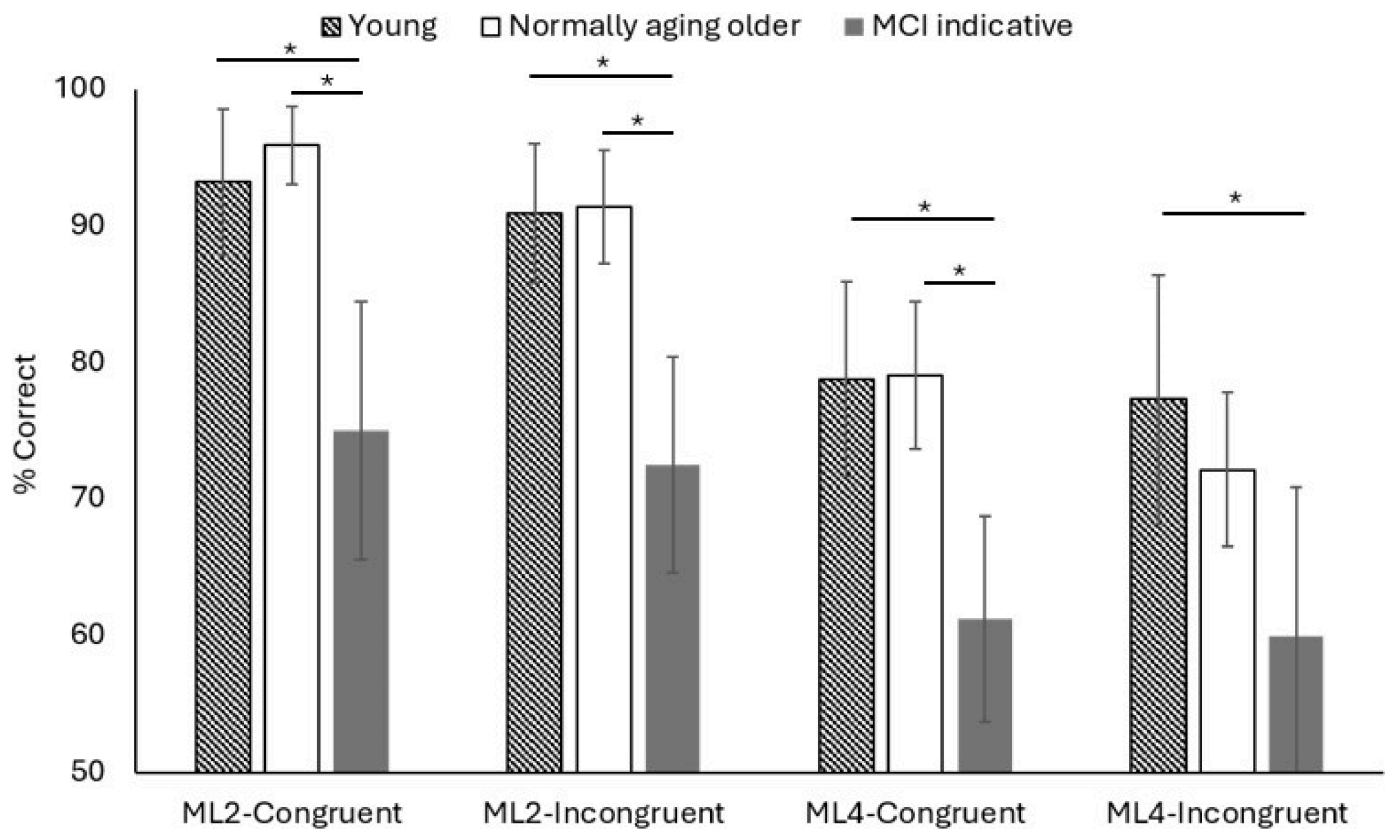
Memory performance (% correct) across participant groups for Experiment 1. Error bars represent ±1.96 standard error. Significant group difference (* *p* < 0.05).

The graphical representation of mean % correct responses as memory performance at ML2 and ML4 for three participant groups is provided in Figure 2.

A mixed ANOVA revealed significant main effects of participant group and memory load, but not congruency (Table 1).

Post hoc pairwise comparison using Bonferroni correction indicated the MCI-indicative group performed significantly worse overall in both ML2 and ML4 trials compared to young and normally aging older participants (*ps* < 0.001). The difference between the normally aging older and young groups was not significant (ML2 *p* = 0.546, ML4 *p* = 0.500).

MCI-indicative group showed significantly worse memory accuracy performance compared to young adults and normally aging older adults in both congruent (*p* < 0.001) and incongruent trials (*p* < 0.001).

All three participant groups performed significantly better in ML2 trials compared to ML4 (*ps* < 0.001). No recency or primacy effect was observed in the memory performance of the three participant groups (*ps* > 0.05).

#### 3.1.2. Reaction Time

**Figure 3:**
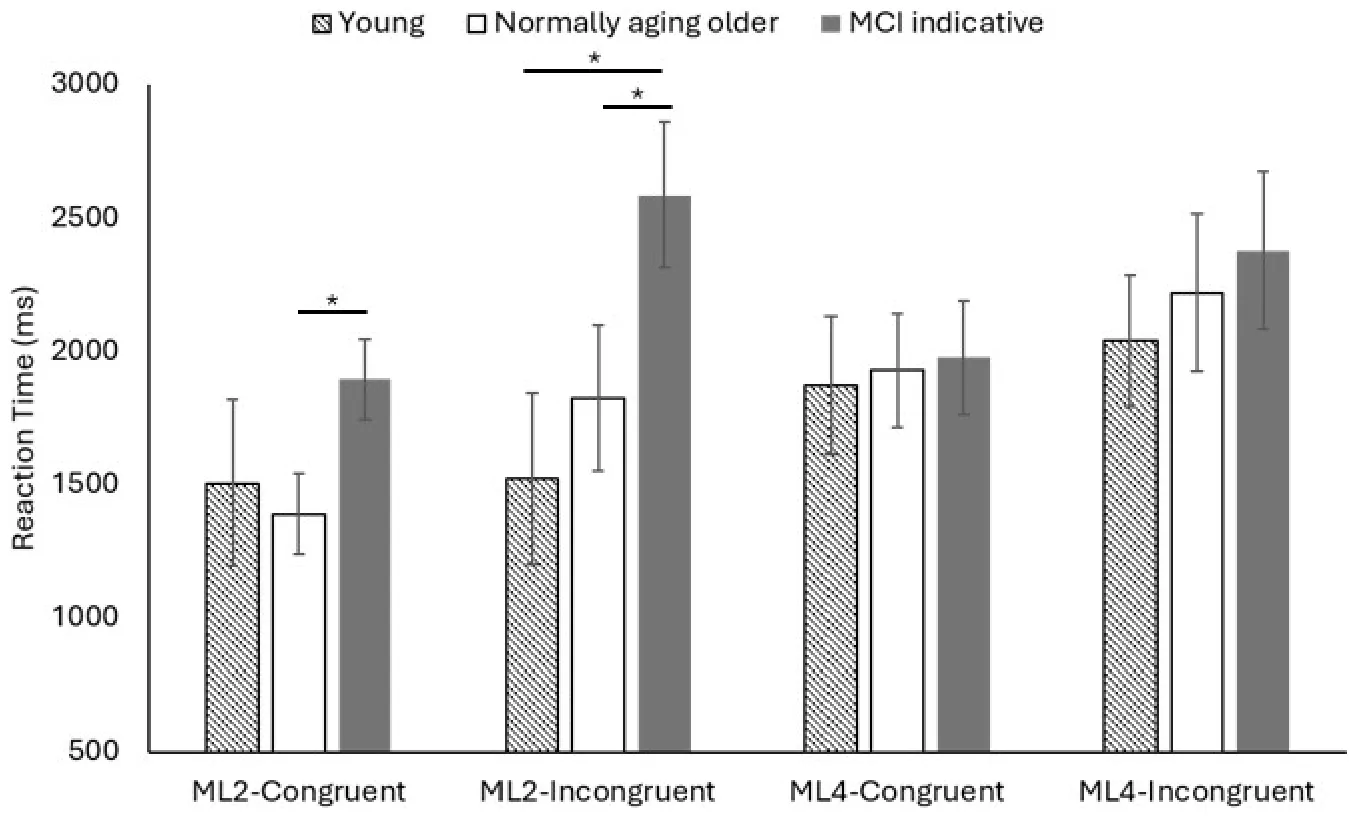
Reaction time (ms) across participant groups for Experiment 1. Error bars represent ±1.96 standard error. Significant group difference (* *p* < 0.05).

The graphical representation of reaction time in ms at ML2 and ML4 trials for three participant groups is provided in Figure 3.

A significant main effect of participant group, memory load and congruency type (congruent/incongruent) on RT was found (Table 2). See Table 2 for a summary of all effects.

The significant main effect of memory load on RT indicated that participants responded significantly slower in the high memory load (ML4) condition compared to the low memory load (ML2) condition. Post hoc Bonferroni-corrected within-groups pairwise comparison revealed that the RT of the young and normally aging older participant groups were significantly longer in ML4 trials compared to ML2 (*ps* < 0.001). This was not shown for the MCI-indicative group (*p* = 0.662). The significant interaction between ML and group indicated that memory load impacted the participant groups differently.

Post hoc Bonferroni-corrected within-group pairwise comparisons showed that the MCI-indicative group was the slowest to respond in ML2 compared to the young (*p* = 0.001) and normally aging older (*p* = 0.002) groups. No significant difference in RT was found between the young and normally aging older groups (*p* = 0.569). At higher memory load (ML4), no significant difference in RT was found between the three groups (*ps* > 0.272).

A significant interaction between participant groups and memory load and participant group and congruency type was found (Table 2). Post hoc Bonferroni-corrected within-group pairwise comparisons revealed that the RT of the normally aging older (*p* < 0.001) and MCI-indicative (*p* < 0.001) groups were significantly longer during incongruent trials compared to congruent trials. This was not true for the young participant group (*p* = 0.409).

Bonferroni-corrected within-group pairwise comparisons revealed that the RT of the MCI-indicative group was significantly longer in incongruent trials compared to the young (*p* = 0.003) and normally aging (*p* = 0.033) groups, while in congruent trials there was no such difference (*ps* > 0.090) in RT.

RT differences for correct and incorrect trials were also analyzed with a mixed-design ANOVA with accuracy type (correct or incorrect) as a within-subjects factor and group as a between-subjects factor. The significant main effect of accuracy indicated that RT was significantly longer for incorrect trials. Post hoc Bonferroni-corrected within-group pairwise comparisons revealed that the RTs of the young (*p* < 0.001) and normally aging older (*p* < 0.001) groups were significantly longer during incorrect trials compared to correct trials. The difference was not significant for the MCI-indicative group (*p* = 0.421).

### 3.2. Experiment 2

#### 3.2.1. Memory Accuracy (%Correct Responses)

**Figure 4:**
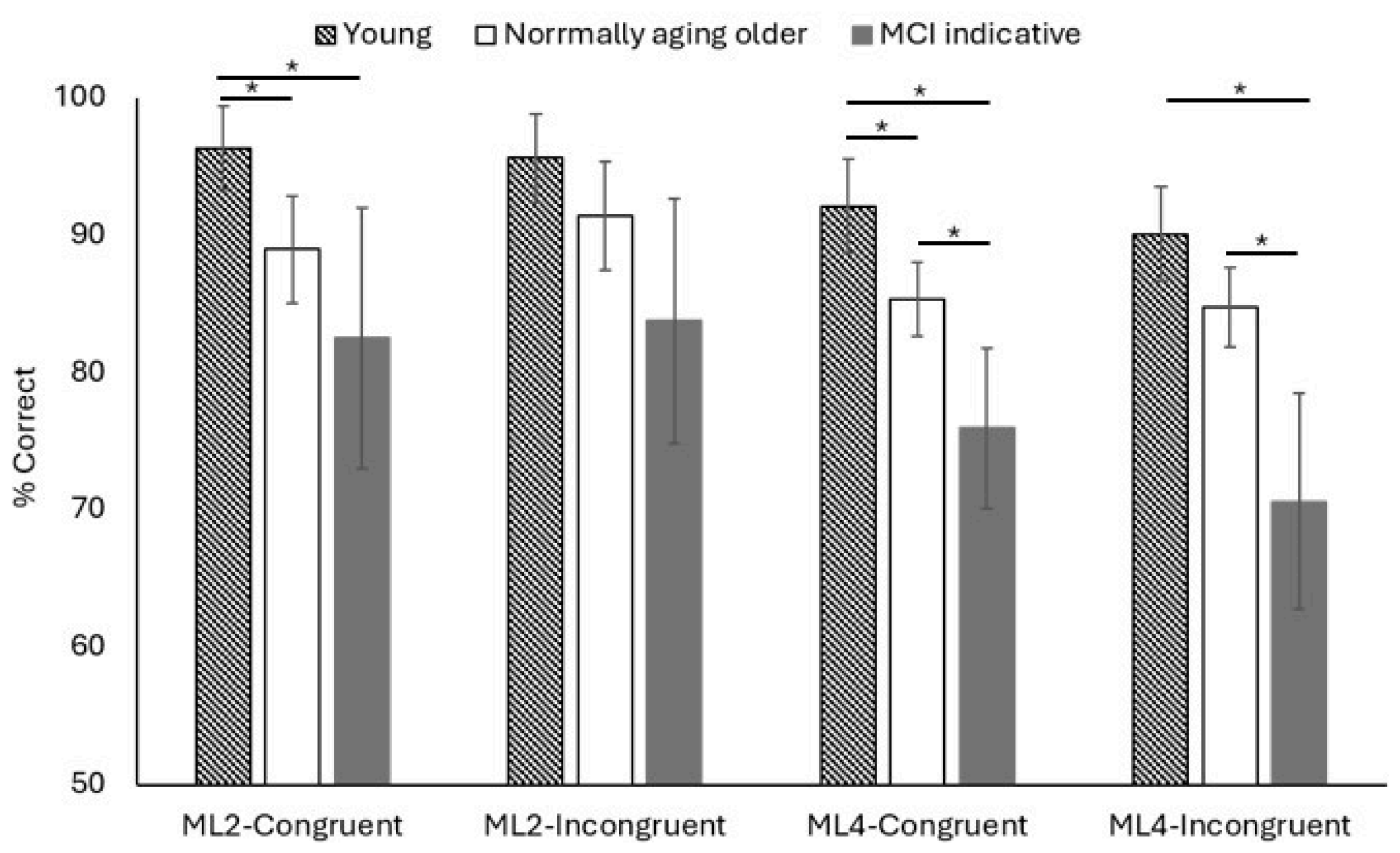
Memory performance (% correct) across participant groups for Experiment 2. Error bars represent ±1.96 standard error. Significant group difference (* *p* < 0.05).

The graphical representation of mean % correct responses as memory performance at ML2 and ML4 for three participant groups is provided in Figure 4.

A significant main effect of participant and memory load on percent correct responses was found. A summary of the main and follow-up analyses on percent correct responses is presented in Table 3.

Post hoc Bonferroni-corrected within-group pairwise comparisons revealed that all three groups performed significantly poorer in ML4 trials compared to ML2 (*ps* < 0.009). Due to the violation of the homogeneity of variances assumption (Levene’s *ps* < 0.001), follow-up analyses at each memory load and congruency condition were conducted using Welch’s ANOVA. Games–Howell post hoc comparisons showed that the young group outperformed the normally aging older group in ML2 (*p* = 0.010) and in ML4 (*p* = 0.006) and MCI-indicative participants in ML2 (*p* = 0.010) and in ML4 (*p* < 0.001). Normally aging older adults performed significantly better only in ML4 (*p* = 0.004) but not in ML2 (*p* = 0.216) compared to the MCI-indicative group.

Games–Howell post hoc comparison also showed that the young group outperformed the normally aging older group in both congruent (*p* = 0.004) and incongruent (*p* = 0.029) trials, and MCI-indicative group in congruent (*p* < 0.001) and in incongruent (*p* < 0.001) trials. The difference between the normally aging older and MCI-indicative groups was also significant in both congruent (*p* = 0.024) and incongruent (*p* = 0.014) trials, in which the normally aging older adults outperformed the MCI-indicative group regardless of congruency.

Young participants showed a recency effect in ML2 trials and were significantly more accurate for the last item (*M* = 1.00, *SD* = 0.00) than for the first item (*M* = 0.94, *SD* = 0.16), *t* (33) = −2.10, *p* = 0.044, *d* = −0.360. For the second memory item, a ceiling effect was also observed; therefore, this result should be interpreted with caution.

#### 3.2.2. Reaction Time

**Figure 5:**
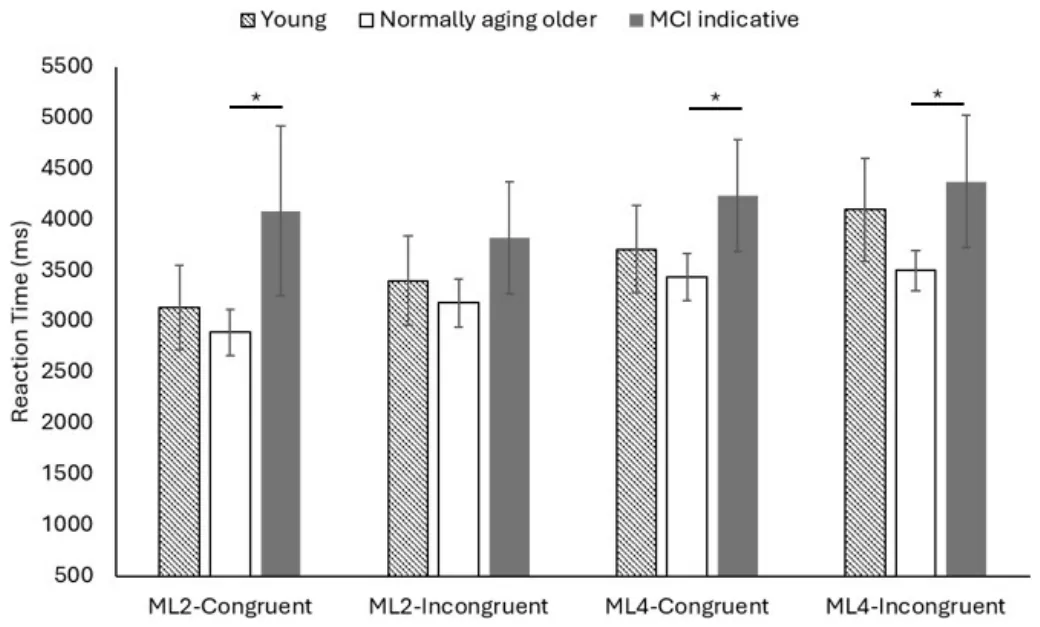
Reaction time (ms) across participant groups for Experiment 2. Error bars represent ±1.96 standard error. Significant group difference (* *p* < 0.05).

The graphical representation of reaction time in ms at ML2 and ML4 trials for three participant groups is provided in Figure 5.

A significant main effect of participant group, memory load and congruency type on RT was found. A summary of all effects, including follow-up Welch tests, is provided in Table 4.

The significant main effect of memory load indicated that RT was significantly longer during the high memory load (ML4) condition compared to low memory load (ML2) condition. Post hoc Bonferroni-corrected within-group pairwise comparisons revealed that the RT of all three participant groups were significantly longer in ML4 trials compared to ML2 (*ps* < 0.010).

Due to the violation of the homogeneity of variances assumption (Levene’s *ps* < 0.037), follow-up analyses at each memory load were conducted using Welch’s ANOVA. A significant main effect of group on RT in ML2 and ML4 trials was found. Games–Howell post hoc comparison showed that the MCI-indicative group took significantly longer compared to the normally aging older group to respond in ML2 (*p* = 0.035) and ML4 (*p* = 0.032) trials. The RT difference between the young and the two older groups was not significant in ML2 (*ps* > 0.194) or ML4 (*ps* > 0.202) trials.

A significant main effect of congruency indicated that RT was significantly longer for incongruent trials. Post hoc Bonferroni-corrected within-group pairwise comparison revealed that the RTs during incongruent trials compared to congruent trials were significantly longer in the young (*p* = 0.002) and the normally aging older (*p* = 0.025) groups. This was not true for the MCI-indicative group (*p* = 0.646).

Welch’s ANOVA (Levene’s test of homogeneity of variances was significant, *ps* < 0.007) revealed a significant main effect of group on RT in congruent and incongruent trials. Games–Howell post hoc comparison showed that the MCI-indicative group took significantly longer to respond compared to the normally aging older group in both congruent (*p* = 0.026) and incongruent (*p* = 0.047) trials. The RT difference between the young and the two older groups was not significant either in congruent (*ps* > 0.220) or incongruent (*ps* > 0.130) trials.

RT differences for correct and incorrect trials were also analyzed with a mixed-design ANOVA, which revealed a significant main effect of accuracy type, indicating that RT was significantly longer for incorrect trials. Post hoc Bonferroni-corrected within-group pairwise comparisons revealed that all three participant groups answered correct trials faster compared to incorrect trials (young *p* < 0.001, normally aging *p* = 0.005, MCI-indicative *p* = 0.002). The significant interaction between accuracy and group suggested that the RT of participant groups may have been impacted differently by the accuracy of their answers.

Follow-up analyses were conducted using Welch’s ANOVA (Levene’s *ps* < 0.012), which revealed a significant main effect of participant group on RT in incorrect and correct trials. Games–Howell post hoc comparison showed that the young group was significantly slower compared to normally aging older group in incorrect (*p* = 0.009) but not in correct (*p* = 0.281) trials. However, in correct trials, the MCI-indicative group was the slowest to respond, taking significantly longer than the normally aging older group (*p* = 0.039). Interestingly, no significant difference was found for RT in incorrect trials between MCI-indicative and normally aging older adult groups (*p* = 0.335). There was no significant effect of group on response bias in Experiments 1 and 2 (*ps* > 0.089).

## 4. Discussion

This study examined the previously unexplored question of whether Stroop-like color incongruency (Experiment 1) and directional incongruency (Experiment 2) influence young, older, and MCI-indicative adults differently during VSTM binding. In Experiment 1, participants knew from the outset that the color information was irrelevant to performing the task and needed to be actively inhibited (Stroop-like task). In Experiment 2, participants did not know the task-irrelevant information (the direction in which the arrow pointed or drifted) until the test display. Response times (RTs) were measured in both experiments.

Across both experiments, the MCI-indicative group showed consistently reduced memory accuracy and slower reaction times (RTs), particularly under the higher memory load (ML4) condition, indicating a general impairment in VSTM binding efficiency with an increased memory load. Importantly, these deficits emerged irrespective of whether task-irrelevant information was known to participants at encoding or introduced only at retrieval, possibly suggesting that MCI-related impairments are not specific to the memory processing stage but reflect a broader disruption of VSTM processing. This is consistent with findings from previous studies showing that VSTM binding accuracy is vulnerable in early cognitive decline [49,50,51,52].

In the color word-location task (Experiment 1), where irrelevant information could be proactively suppressed at encoding, cognitively normal older adults performed comparably to younger adults. In contrast, significantly reduced performance accuracy was observed for cognitively normal older adults (vs. young adults) in the directional binding task, where task-irrelevant information became relevant only at retrieval. A plausible explanation for this finding may be that proactive inhibitory mechanisms are relatively preserved in normal aging. In the MCI-indicative group, however, impairments were evident across both tasks, indicating a more pervasive disruption of inhibitory processes, which needs further investigation.

In the directional binding task (Experiment 2), cognitively normal older group performance deficits were limited to reduced accuracy, without significant slowing in RT, possibly suggesting an early impairment in binding processes. In contrast, MCI-indicative group performance deficits were limited to both reduced accuracy and increased RT, indicating combined deficits in binding and processing efficiency.

Stimulus congruency did not affect memory accuracy. However, incongruent trials were associated with slower RTs. This dissociation indicates that incongruency primarily affects processing efficiency rather than memory fidelity. Given that inhibitory control is closely associated with prefrontal functioning [23] and declines with both aging and MCI, the observed deficits likely reflect deterioration in executive control systems rather than purely perceptual limitations. Interestingly, whether the stimuli were congruent or incongruent influenced only how quickly the MCI-indicative group responded during the color word-location binding task; Incongruency produced significantly longer reaction times, possibly suggesting incongruency mainly influenced processing speed rather than memory accuracy. Our observed findings on the differential effect of stimulus congruency across participant groups in Experiment 1 were not shown in Experiment 2. Specifically, in Experiment 1, a statistically significant Group × Congruency interaction was observed (Table 2), indicating that stimulus congruency affected the participant groups differently. Experiment 2 did not show this as a significant Group × Congruency interaction, or a significant Group × Congruency × Memory Load interaction (Table 4).

An aging effect in memory accuracy was observed for the directional binding task (Experiment 2) in both low (ML2) and high (ML4) memory load, and in congruent and incongruent trials, suggesting a general age-related decline in VSTM binding ability for direction of pointing and drift for moving arrows. However, this decline was not reflected in slower RT (i.e., young and normally aging older adults had similar RTs).

An MCI effect was also found in directional binding, with the MCI-indicative group performing significantly worse than the normally aging older group, but only at high memory load (ML4). This accuracy impairment was accompanied by longer RTs, implying that the MCI-indicative group not only performed less accurately but also needed more time to process and respond, showing both memory accuracy and processing-speed deficits.

In the color word-location task (Experiment 1), impaired performance for the MCI-indicative group was also found to be independent of memory load or color congruency, with longer RTs in ML2 and in incongruent trials compared to the young and normally aging older groups. Performance comparison between young and cognitively normal older adults suggested no significant effect of normal aging in the color word-location test with both memory accuracy and RT data. The absence of significant reaction-time differences between groups even under high memory load (ML4) may reflect the increased difficulty of the task for all participants. As memory load increased, both young and cognitively healthy older adults showed longer RTs, suggesting greater processing demands. The impaired performance of the MCI-indicative group may also be explained by a reduced ability to access the ‘memory source’ during retrieval as a consequence of failing to prime bound features [40]. This observed impaired performance of the MCI-indicative group could also stem from a binding deficit during encoding, as well as a retrieval deficit when test cues are presented, though the present study was unable to differentiate between these two stages. Furthermore, MCI-related effects may also be due to differences in the rate of information processing, with faster processing associated with better encoding of items in VSTM [53,54].

In the directional binding task (Experiment 2), all three groups performed significantly better at low vs. high memory loads, which was also accompanied by longer RTs at high memory load. Similarly, in the color word-location task, all three groups performed significantly better at low memory load (ML2) compared to high memory load (ML4); however, only the young and the normally aging older group responded more quickly to ML2 vs. ML4 trials, with no such difference in RT between MLs for the MCI-indicative group. Furthermore, in the color word-location test, the MCI-indicative group responses were significantly slower compared to the other two groups, but only in ML2 trials. With ML4, the RT of the three participant groups were not significantly different. This may be explained by a cognitive slowing of the MCI-indicative group that was detectable even at the less demanding memory load. When task difficulty increased (in ML4), all three groups were equally constrained by task complexity.

The drop in memory accuracy performance across all three participant groups from ML2 to ML4 may be due to VSTM capacity saturation. As reported in classical work, VSTM capacity is thought to be limited to around four items, depending on stimulus complexity and familiarity [2,55]. When unfamiliar items are used, capacity is even lower, often ~2 items [56,57].

In the directional binding task, interestingly, the young and normally aging older groups took significantly longer to respond to incongruent trials compared to congruent trials, while congruency did not have an impact on RT for the MCI-indicative groups. The effect of congruency can depend on attentional load. A previous study [58] showed that increasing VSTM load reduced the interference caused by incongruent distractors. This suggests that a taxed VSTM can be less susceptible to outside interference. During the directional binding experiment, participants were required to remember both the direction that the arrow pointed and the direction of its drift. In contrast, during the color word-location task, participants were instructed to ignore the color of the text and focus only on the word and its location from the outset. This reduced VSTM load (with possible increased susceptibility to disruption from congruency interference) may explain why the two older groups (normally aging and MCI-indicative) took significantly longer to respond to incongruent trials compared to congruent trials in the color word-location task. According to the load theory [59,60,61] the extent to which irrelevant distractors are processed depends on the perceptual load of the primary task. When perceptual load is low, spare attentional capacity ‘spills over’ to irrelevant stimuli, increasing distraction; when load is high, attentional resources are fully engaged, reducing distractor processing.

Additionally, in the color word-location task, older groups were impacted more by stimulus incongruency, as reflected in longer RTs. This is in line with the study by Hofeijzers et al. [17] who found older adults to be more impacted by incongruency, in which they performed significantly better in a congruent vs. incongruent color-object recognition task. However, in the current study, this was only reflected in RT but not in memory performance. Conti et al. [18] also found that participants with dementia had longer RTs in an incongruent condition compared to a congruent condition. RT differences between conditions reflect the disruptive influence of incongruent features and the intrusion of irrelevant stimulus features [19] that may disproportionately impact older adults.

Goldfarb and Treisman [19] found a binding congruency effect in young participants with digit-physical size and word-font color binding tasks. Participants committed more memory errors when incongruent features were bound together, such that incongruent features were often replaced with congruent features when they answered the task question. However, their study had a small sample size (*N* = 8).

In our study, both tasks primarily engage explicit (goal-directed control), top-down inhibitory control, rather than implicit (automatic and unconscious suppression) processes. The color word-location task requires intentional suppression of a prepotent but irrelevant stimulus dimension, consistent with Stroop-like interference effects [12,13].

In the directional binding task, participants needed to regulate attention and suppress competing feature representations under probe uncertainty, drawing on executive control mechanisms [62,63]. As such, conflict resolution is linked to prefrontal functioning [64,65] and as inhibitory control declines with age and in mild cognitive impairment [23,25,64,66] age-related differences in these tasks likely reflect deterioration in explicit executive inhibition rather than automatic filtering deficits.

The directional binding paradigm differs from spatial Stroop, and arrow-based Simon and flanker tasks [67,68,69,70,71,72] in that the conflict is embedded within a sequential working memory framework rather than probed at response selection. Whereas these traditional tasks involve immediate responses to single stimuli or stimulus arrays, indexing interference between competing stimulus features or response tendencies, the current design requires maintaining and binding multiple stimulus features (drift and direction) across time, followed by delayed, feature-specific retrieval. This approach therefore captures not only interference control but also its interaction with working memory and retrieval processes.

In the current study, all three participant groups responded slowly during incorrect vs. correct trials in the directional binding task, as one would expect [73]. An accuracy vs. group interaction was found in RT data, suggesting that extended RTs for incorrect responses impacted participant groups differently. Interestingly, the younger group took longer to answer during incorrect trials compared to the normally aging older group, while the MCI-indicative group took longer to answer during correct trials compared to the normally aging older group in the directional binding task. In the color word-location task, only the young and normally aging older groups took longer to answer during the incorrect trials compared to the correct trials. Interestingly, the MCI-indicative group answered with similar speed regardless of whether they were correct or not in the color word-location task. In VSTM tasks, longer RT in incorrect trials compared to correct ones may reflect increased cognitive demand and uncertainty [74]. This effect may be due to weaker or noisier memory traces, which slow the decision-making process. Additionally, incorrect responses may result from difficulty in resolving feature bindings, requiring more time to process ambiguous or conflicting information. Studies have also shown that incorrect trials are associated with slower RTs due to elevated internal monitoring and increased cognitive control [75]. These findings suggest that longer RTs on incorrect trials may serve as indirect markers of impaired binding or degraded memory representations. The lack of RT differentiation (between correct and incorrect trials) in the MCI-indicative group may reflect impaired error awareness or reduced performance monitoring in MCI, potentially due to early impairment of cognitive control [34,76]. Alternatively, the MCI-indicative group may operate with lower decision thresholds or reduced capacity to adjust response strategies based on task difficulty, as proposed by drift-diffusion models [77]. These findings suggest that MCI may be associated not only with memory deficits but also with disruptions in the metacognitive processes (knowledge, control, and management of one’s own cognitive processes) that support adaptive decision-making.

The following limitations of our study should be noted. First, it was a cross-sectional study; hence, our data do not allow us to determine whether the observed effects predict future MCI. Also, the causal direction between inhibitory decline and information processing speed in explaining the aging effects could not be determined. Second, although the sequential presentation paradigm combined with articulatory suppression was used to enhance internal validity, findings obtained under these laboratory conditions may not fully generalize to real-world contexts. Third, the MCI-indicative group had a relatively smaller sample size.

## 5. Conclusions and Future Directions

MCI-indicative participants exhibited greater impairment in color word-location and directional binding compared to normally aging older adults. Stimulus incongruency did not have a significant impact on memory performance for any group, but elicited longer RT. RTs appear to capture variance not reflected in accuracy measures and may warrant further evaluation in clinically diagnosed samples. Our objective was to investigate individuals who screened positive for MCI and hence were at greater risk of clinical MCI, rather than to establish findings that are specific to clinically diagnosed MCI cases. Future longitudinal research may evaluate how these findings predict subsequent cognitive decline, establish their diagnostic utility for identifying clinical MCI compared with standard tests, and determine their generalizability to clinical populations by including individuals with a confirmed clinical diagnosis of MCI. It was not possible, in this study design, to reveal whether the lower performance in the MCI-indicative group is due to impaired memory binding, the additional irrelevant information during presentation (requiring inhibition) of the stimuli or inherent differences between the MCI-indicative and control groups. Future research may expand the color word-location test by comparing memory binding performance between colored (congruent and incongruent) and non-colored stimuli to explore if the irrelevant information (color of the text) reduces memory binding ability. Similarly, single-direction control tests (e.g., testing memory for a static arrow and moving square) for directional binding could be explored.

With global populations aging rapidly, there is a growing need to identify sensitive cognitive markers that can detect subtle decline before dementia develops. MCI represents a critical transitional stage between healthy aging and dementia, where cognitive changes are measurable but not yet severe, making it essential to develop tools capable of capturing the earliest deficits, and making congruency a potentially useful manipulation for probing early deficits.

## Figures and Tables

**Table 1:** Summary of mixed ANOVA results for percentage correct responses in Experiment 1.

Effect	F (df1, df2)	*p*-Value	Effect Size (η^2^_p_)
Participant group	17.48 (2, 111)	<0.001	0.24
Memory load (ML)	80.81 (1, 111)	<0.001	0.42
Congruency	2.84 (1, 111)	0.095	0.03
Group × ML	1.05 (2, 111)	0.354	0.02
ML × Congruency	0.002 (1, 111)	0.968	<0.001
Group × Congruency	0.64 (2, 111)	0.530	0.01
ML × Congruency × Group	0.13 (2, 111)	0.879	0.002

**Table 2:** Summary of ANOVA results for RT in Experiment 1.

Effect	F (df1, df2)	*p*-Value	Effect Size (η^2^_p_)
Main RT analysis			
Participant group	3.35 (2, 111)	0.039	0.06
Memory load (ML)	18.64 (1, 111)	<0.001	0.14
Congruency	26.05 (1, 111)	<0.001	0.19
Group × ML	5.61 (2, 111)	0.005	0.09
Group × Congruency	3.61 (2, 111)	0.030	0.06
ML × Congruency	0.73 (1, 111)	0.393	0.01
ML × Congruency × Group	1.32 (2, 111)	0.273	0.02
RT by accuracy (correct vs. incorrect)			
Accuracy	24.81 (1, 100)	<0.001	0.20
Participant group	0.22 (2, 100)	0.805	0.004
Accuracy × Group	2.42 (2, 100)	0.094	0.046

**Table 3:** Summary of mixed ANOVA results for percentage correct responses in Experiment 2.

Effect	F (df1, df2)	*p*-Value	Effect Size (η^2^_p_)
Main ANOVA			
Participant group	15.99 (2, 112)	<0.001	0.22
Memory load (ML)	35.88 (1, 112)	<0.001	0.24
Congruency	0.44 (1, 112)	0.507	0.004
Group × ML	1.68 (2, 112)	0.192	0.03
ML × Congruency	2.85 (1, 112)	0.094	0.03
Group × Congruency	0.65 (2, 112)	0.522	0.01
ML × Congruency × Group	0.40 (2, 112)	0.670	0.01
Welch’s ANOVA			
Group (ML2)	8.18 (2, 45.34)	<0.001	0.27
Group (ML4)	14.61 (2, 44.46)	<0.001	0.40
Group (Congruent)	12.54 (2, 45.82)	<0.001	0.35
Group (Incongruent)	10.52 (2, 44.75)	<0.001	0.32

**Table 4:** Summary of ANOVA results for RT in Experiment 2.

Effect	F (df1, df2)	*p*-Value	Effect Size (η^2^_p_)
Main RT analysis			
Participant group	5.54 (2, 112)	0.005	0.09
Memory load (ML)	57.24 (1, 112)	<0.001	0.34
Congruency	5.56 (1, 112)	0.020	0.05
Group × ML	1.76 (2, 112)	0.176	0.031
Group × Congruency	2.62 (2, 112)	0.077	0.05
ML × Congruency	0.80 (1, 112)	0.372	0.01
ML × Congruency × Group	2.93 (2, 112)	0.058	0.05
Welch’s ANOVA			
Group (ML2)	3.68 (2, 40.65)	0.034	0.15
Group (ML4)	4.55 (2, 40.28)	0.017	0.18
Group (Congruent)	4.06 (2, 41.99)	0.024	0.16
Group (Incongruent)	4.58 (2, 39.16)	0.016	0.19
RT by accuracy (correct vs. incorrect)			
Accuracy	41.99 (1, 106)	<0.001	0.28
Participant group	5.83 (2, 106)	0.004	0.10
Accuracy × Group	3.55 (2, 106)	0.032	0.06
Welch’s ANOVA			
Group (Incorrect trails)	5.60 (2, 35.54)	0.008	0.24
Group (Correct trails)	4.06 (2, 40.39)	0.025	0.17

## Data Availability

All data supporting the findings of this study are included within the manuscript. Additional datasets are available from the corresponding author upon reasonable request due to privacy reasons.

## Abbreviations

The following abbreviations are used in this manuscript:

ACE-III	Addenbrooke’s Cognitive Examination III
ANOVA	Analysis of variance
MCI	Mild cognitive impairment
ML2	Memory load 2
ML4	Memory load 4
MoCA	Montreal Cognitive Assessment
RT	Reaction time
VSTM	Visual short-term memory
